# Severe Weight Loss and Its Association with Fatigue in Old Patients at Discharge from a Geriatric Hospital

**DOI:** 10.3390/nu11102415

**Published:** 2019-10-10

**Authors:** Kristina Franz, Lindsey Otten, Ursula Müller-Werdan, Wolfram Doehner, Kristina Norman

**Affiliations:** 1 Charité—Universitätsmedizin Berlin, Freie Universität Berlin, Humboldt Universität zu Berlin and Berlin Institute of Health, Research Group on Geriatrics, Working Group Nutrition and Body Composition, Reinickendorfer Str. 61, 13347 Berlin, Germany; lindsey.otten@charite.de (L.O.); kristina.norman@charite.de (K.N.); 2 German Institute of Human Nutrition Potsdam - Rehbrücke, Department of Nutrition and Gerontology, Arthur—Scheunert—Allee 114-116, 14558 Nuthetal, Germany; 3 Protestant Geriatric Center Berlin, Reinickendorfer Str. 61, 13347 Berlin, Germany; ursula.mueller-werdan@charite.de; 4 Charité—Universitätsmedizin Berlin, Freie Universität Berlin, Humboldt Universität zu Berlin and Berlin Institute of Health, Research Group on Geriatrics, Reinickendorfer Str. 61, 13347 Berlin, Germany; 5 Charité—Universitätsmedizin Berlin, corporate member of Freie Universität Berlin, Humboldt Universität zu Berlin and Berlin Institute of Health, BCRT—Berlin Institute of Health Centre for Regenerative Therapies, Föhrer Str. 15, 13353 Berlin, Germany; wolfram.doehner@charite.de; 6 DZHK—German Centre for Cardiovascular Research, Partner Site Berlin, Potsdamer Str. 58, 10785 Berlin, Germany; 7 University of Potsdam, Institute of Nutritional Science, Arthur—Scheunert—Allee 114-116, 14558 Nuthetal, Germany

**Keywords:** malnutrition, involuntary weight loss, post-hospital syndrome, fatigue, old adults

## Abstract

Although malnutrition is frequent in the old, little is known about its association with fatigue. We evaluated the relation of self-reported severe weight loss with fatigue and the predictors for fatigue in old patients at hospital discharge. Severe weight loss was defined according to involuntary weight loss ≥5% in the last three months. We determined fatigue with the validated *Brief Fatigue Inventory* questionnaire. The regression analyses were adjusted for age, sex, number of comorbidities, medications/day, and BMI. Of 424 patients aged between 61 and 98 y, 34.1% had severe weight loss. Fatigue was higher in patients with severe weight loss (3.7 ± 2.3 vs. 3.2 ± 2.3 points, *p* = 0.021). In a multinomial regression model, weight loss was independently associated with higher risk for moderate fatigue (OR:1.172, CI:1.026–1.338, *p* = 0.019) and with increased risk for severe fatigue (OR:1.209, CI:1.047–1.395, *p* = 0.010) together with the number of medications/day (OR:1.220, CI:1.023-1.455, *p* = 0.027). In a binary regression model, severe weight loss predicted moderate-to-severe fatigue in the study population (OR:1.651, CI:1.052-2.590, *p* = 0.029). In summary, patients with self-reported severe weight loss at hospital discharge exhibited higher fatigue levels and severe weight loss was an independent predictor of moderate and severe fatigue, placing these patients at risk for impaired outcome in the post-hospital period.

## 1. Introduction

In the old, malnutrition is a common occurrence in patients with acute and chronic disease or injury, where it is in part triggered by disease-associated inflammatory mechanisms. Furthermore, anorexia of aging, which results in a decline in appetite and nutritional intake, occurs frequently in older people affecting approximately one-third of older men and women in long-term facilities and rehabilitation or geriatric settings [1], and is an important contributor to malnutrition. The mechanism of anorexia of aging is complex, involving the altered response of some brain areas, such as the hypothalamus, to changes in circulating hormones, adipokines, and nutrients [2]. Malnutrition is reflected by weight loss and predominantly associated with loss of muscle mass [3,4,5] in old patients, increasing the risk for sarcopenia (e.g., the age-associated loss of muscle mass and function). Loss of weight and muscle mass are predictive of reduced functional outcomes and decreased quality of life [6]. The prevalence of malnutrition varies depending on the applied screening tool [7,8] but occurs in up to 50% of old patients at hospital admission [7]. 

Malnourished patients have more post-operative infectious and non-infectious complications, a higher non-elective hospital readmission rate, and increased morbidity and mortality after hospital discharge [6,9]. Patients who are malnourished at discharge are most vulnerable for impaired outcome in the transition period from hospital to the ambulatory setting. Moreover, older adults have reduced compensatory mechanisms after weight loss and in particular, regain of fat-free mass is hampered. Loss of weight and muscle mass as well as reduced nutritional intake have been linked to fatigue. Fatigue has been described as an overwhelming exhaustion affecting the ability to carry out physical and mental activities [10]. It is related to an impairment of a variety of functional parameters and has debilitating effects on quality of life [11]. It most likely impedes convalescence after hospitalization and it predisposes to negative outcomes [10]. Although fatigue is most frequently associated with cancer where it has been associated with an impaired nutritional status [12,13], it is prevalent in other chronic diseases as well [14]. Fatigue is highly frequent in the old, but its complex and multifactorial etiology is not well understood [15]. However, fatigue has been proposed as a key component or a precursor of the frailty syndrome [10,11,16], and most likely reflects the decreased reserve and impaired resistance to internal and external stressors, which leads to higher vulnerability to negative outcomes in frail individuals [11]. 

Altogether, relatively little is known about the association between malnutrition (e.g., lack of essential nutrients, progressive loss of weight, and muscle mass) and fatigue in the old. The objective of this cross-sectional study in old patients was to identify and describe (i) the relation of self-reported severe involuntary weight loss as a marker of catabolic malnutrition and fatigue, as well as (ii) the predictors for fatigue at hospital discharge.

## 2. Materials and Methods 

### 2.1. Study Protocol and Recruited Patients

The study was designed as a prospective cross-sectional trial from the Department of Geriatrics at the Charité–University of Medicine Berlin. All participants signed a written informed consent prior to their inclusion in the study. The trial was conducted in accordance with the Declaration of Helsinki of 1975 and with the approval of the Ethics Committee of the Charité–University Medicine Berlin (clinicaltrials.gov identification code: NCT03126500). 

Patients were consecutively recruited at discharge from an acute geriatric clinic. Exclusion criteria were < 60 years of age, impaired cognitive status according to the *Mini-Mental State Examination* assessed by the neuropsychologist (MMSE cut off < 24 points), dementia or ongoing delirium, palliative care for end-stage disease, edema and ascites, or no understanding of the German language. Of eligible patients, 26.8 percent declined participation in the study.

Age, sex, clinical data (type of principal diagnosis at discharge, number of comorbidities, number of medications/day (not including nutritional supplements), and length of hospital stay at discharge) were recorded. The ability to carry out activities of daily living was assessed with the Barthel Index questionnaire (Activities of daily living, ADL, lower values indicate higher dependence) [17].

Further tests include isometric handgrip strength (dynamometry, JAMAR, Preston Bissell Healthcare Co., Jackson, MI, USA) as well as gait speed by a 4 m walking test.

### 2.2. Anthropometric Measurements and Detection of Severe Weight Loss

Weight was measured at standardized conditions in light clothes with no shoes with a portable electronic scale to the nearest 0.1 kg (seca 910; seca, Hamburg, Germany) and height to the nearest 0.01 m was measured with a stadiometer (seca 220 telescopic rod, seca, Hamburg, Germany). Weight and height were used to calculate body mass index (BMI, weight/height^2^, kg/m^2^). We used 4 categories of BMI stratified according to age as suggested by the National Academy of Science, indicating underweight, normal weight, overweight, and obesity (≤65 y: <23 kg/m^2^, 23–28 kg/m^2^, >28–30 kg/m^2^, ≥30 kg/m^2^ as well as >65 y: <24 kg/m^2^, 24–29 kg/m^2^, >29–30 kg/m^2^, ≥30 kg/m^2^) [18]. 

Self-reported weight changes in the last three months were recorded. Patients who had lost ≥5% weight during the last three months [19] were compared to those with weight loss < 5% or without any weight loss in the previous three months.

### 2.3. Assessment of Fatigue

Fatigue was estimated using the *Brief Fatigue Inventory* (BFI, The University of Texas M. D. Anderson Cancer Center, Houston, Texas, 1997) questionnaire [20], which was originally developed for cancer patients, but has been shown to be a valid instrument with established psychometric properties in the old [21]. It consists of 10 questions assessing the presence of fatigue (fatigue right now, usual fatigue in the last 24 h, and worst fatigue), as well as fatigue-related impairment of general activity, mood, walking ability, normal working, relationships with others, and vitality. A score for each item was calculated and the total fatigue score is between 0 to 10 (higher score indicates higher intensity of fatigue). Cut-offs for fatigue severity were calculated according to validated fatigue ratings (no fatigue: 0 points; mild fatigue: 1 to 3 points; moderate fatigue: 4 to 6 points; severe fatigue: 7 to 10 points) [22]. 

For the binary logistic regression analyzing the predictors of fatigue, the fatigue severity was categorized as a binary variable (no/mild fatigue: <4 points; moderate/severe fatigue: ≥4 points) for clearer descriptive specifications of the study population. 

### 2.4. Data Analysis

Statistical analysis was carried out using the statistical software SPSS © (IBM version 25, SPSS Inc. Chicago, IL, USA). Descriptive analyses were given as mean and standard deviation (SD) or total number and percentage (%). 

Clinical data and fatigue scores were compared between the groups using the Student´s t-test for metric parameters or the chi-squared test for nominal parameters. Comparisons of weight loss according to severity of fatigue (no/mild/moderate/severe) were performed with the ANOVA as well as with the Bonferroni post hoc test. Pearson’s correlation coefficients were calculated to identify the relationship between weight loss and fatigue score. 

For the evaluation of the association between weight loss and fatigue, two regression models were used: i) multinomial regression (model 1) in all patients who lost weight using all fatigue categories (no; mild; moderate; severe), and ii) binary logistic regression (model 2) in the overall sample size using the binary fatigue variable (no/mild; moderate/severe). Both regression models were adjusted for sex, age, number of comorbidities, and medications as well as BMI. Statistical significance was set a priori at *p* < 0.05. Column plots show mean and SD and were created with Graph Pad Prism 7.0.

## 3. Results

### 3.1. Participant Characteristics and Weight Loss at Hospital Discharge

A total number of 424 hospital patients were included in this trial. Of all patients, 278 (67.6%) reported weight loss within three months, with 140 (34.1%) patients exhibiting severe loss of weight ≥5%, with a mean weight loss of −10.3 ± 4.8% (vs. −1.3 ± 1.6%, *p* < 0.001). Demographic, clinical, nutritional, and functional parameters are reported in Table 1. The age in the overall sample ranged from 61 to 98 years (≤ 65 y: 3.8% of the patients; > 65 y: 96.2%). The age distribution in the study was as follows: 25% of the patients were 61–74 y, 25% of the patients were 75–78 y, 25% of the patients were 79–82 y, and 25% of the patients were 83–98 y. Mean age was lower in patients with severe weight loss and sex distribution did not differ between the two patient groups. As anticipated, we observed greater functional decline in activities of daily living in patients with severe weight loss, although grip strength and gait speed were comparable. Of the total number of patients, the BMI distribution was as follows: 45.5% of patients were underweight, 34.4% of patients were normal weight, 4.1% of patients were overweight, and 16.0% of patients were obese. The majority of all patients had orthopedic disorders, followed by heart disease. Patients aged > 65 years who exhibited severe weight loss, had a higher number of medications per day and an increased length of hospital stay. 

### 3.2. Higher Fatigue in Patients with Severe Weight Loss at Hospital Discharge

**Figure 1:**
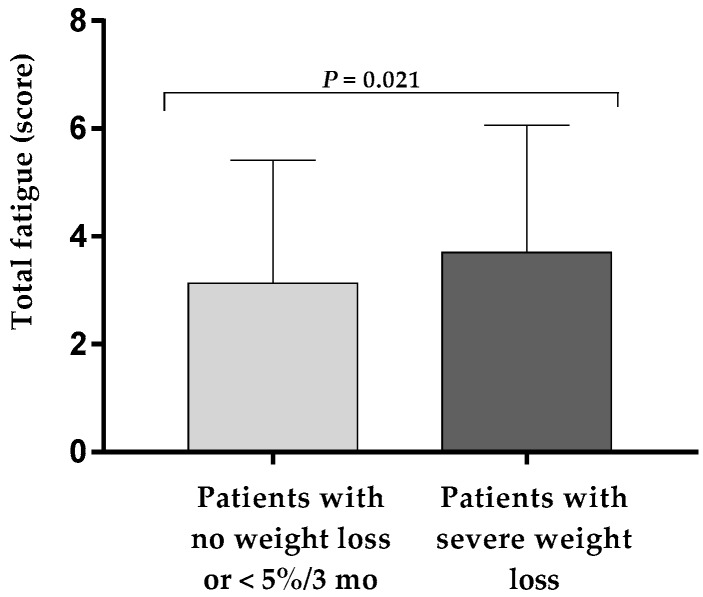
Total fatigue score according to self-reported severe weight loss at hospital discharge. The column plot represents mean and SD.

In the overall sample size, 55.5% of the patients had mild fatigue, followed by 28.9% with moderate, and 7.6% with severe fatigue. The usual and worst fatigue score (Table 2), as well as total fatigue score (Figure 1), ranging from 0 to 10 points, were higher in patients with severe weight loss, and the total fatigue score was significantly higher in women with severe weight loss compared to men with severe weight loss (3.8 ± 2.3 points vs. 3.1 ± 2.2 points, *p* = 0.033).

**Figure 2:**
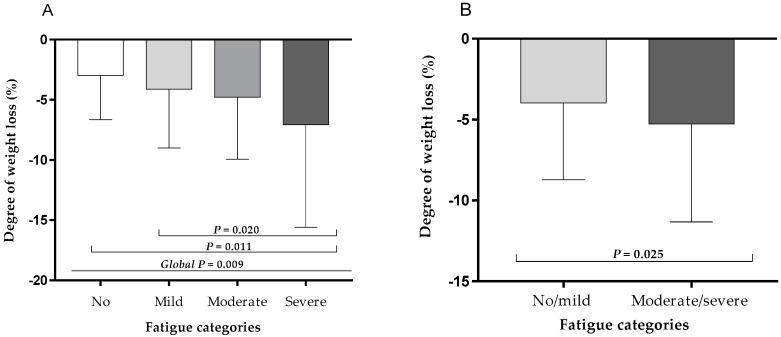
Degree of self-reported weight loss in old patients stratified according to four fatigue categories (**A**) as well as two fatigue categories (**B**) at hospital discharge. Column plots represent mean and SD maximum values.

Moreover, the ANOVA showed a significant difference in the degree of weight loss (%) between the four fatigue severity groups (no, mild, moderate, severe) as shown in Figure 2A. The highest degree of weight loss was seen in patients with severe fatigue compared to mild fatigue and to patients without fatigue (Figure 2A). When classifying patients into two fatigue severity groups (no/mild vs. moderate/severe), patients with moderate/severe fatigue had lost significantly more weight in the last three months compared to the others (Figure 2B). 

Furthermore, we obtained weak inverse correlations between weight loss (%) and individual fatigue items, such as fatigue right now (r = −0.113, *p* = 0.023), usual fatigue (r = −0.132, *p* = 0.008), worst fatigue (r = −0.145, *p* = 0.004), impairment on general activity (r = −0.118, *p* = 0.017), mood (r = −0.118, *p* = 0.018), relationships with others (r = −0.142, *p* = 0.004), and vitality (r = −0.140, *p* = 0.005). The total fatigue score inversely correlated with weight loss in the overall sample size (r = −0.154, *p* = 0.002). 

### 3.3. Association of Severe Weight Loss with Fatigue Severity at Hospital Discharge

In the multinomial regression adjusted for age, sex, number of comorbidities and medications, and BMI shown in Table 3 (model 1), higher weight loss in the last three months (introduced as a metric variable) had a negative effect on moderate fatigue and on severe fatigue in patients with weight loss. Age, sex, number of comorbidities, and BMI had no significant association with fatigue severity.

In the next step, age, sex, number of comorbidities and medications, BMI and malnutrition were entered into a binary logistic regression model shown in Table 4 (model 2), in which severe weight loss emerged as a significant risk factor for moderate/severe fatigue in the overall study population. In contrast, age, sex, clinical parameters, and BMI had no significant effect on fatigue in this study population. 

## 4. Discussion

Our study shows that approximately a third of old patients at discharge reported severe weight loss, and these patients had significantly higher fatigue levels than patients with less or no weight loss. Multinomial regression analysis also showed an association between the degree of weight loss and moderate as well as severe fatigue in patients who exhibited weight loss within three months. In these old patients, self-reported severe weight loss emerged as a predictor for moderate-to-severe fatigue independent of age, sex, number of comorbidities as well as medications/day, and BMI. 

Severe involuntary weight loss reflects a progressive catabolic state and it is a strong independent predictor of impaired outcome [4]. It is related to functional limitations and to a decreased bone and muscle mass [6], immune deficit, impaired wound healing, delayed recovery from surgery [23], increased hospitalization, and readmission rates [9]. Many studies have shown that malnutrition and in particular weight loss, is predictive for mortality e.g., in patients with cancer [24], in old patients discharged from hospital [25], and old patients in nursing homes [26]. 

Although malnutrition is frequent in vulnerable, multi-morbid patients aged ≥ 60 y [27], to our knowledge, only a few studies have addressed the association of impaired nutritional status and fatigue in the old. Gingrich et al. recently showed that more than half of cachectic old study patients exhibited fatigue according to the FACIT-F questionnaire (Functional Assessment of Chronic Illness Therapy Fatigue Scale) [28]. In patients with Parkinson’s disease, fatigue was higher in malnourished patients or patients at risk of malnutrition [14]. The mechanisms underlying fatigue in the old are not well understood [10] but are complex and multidimensional [29]. Fatigue can occur as an acute or chronic state, most often observed in specific medical disorders such as cancer, heart failure, and stroke [30,31]. Impaired nutritional status has frequently been linked to fatigue in cancer patients [32,33]. Stobäus et al. moreover showed that recent protein intake in cancer patients is associated with higher fatigue, implying that not only nutritional status but also acute dietary intake has an effect on fatigue [34]. Multi-morbid, old patients have increased nutrient requirements to maintain homeostasis, and physical fatigue in malnourished patients might be partly due to the result of an imbalance or lack of energy or nutrients [35], although our cross-sectional study design does not allow us to conclude on cause or effect. Moreover, the relationship between impaired nutritional status and fatigue is likely to be interrelated, as fatigue indirectly may limit dietary intake by affecting appetite and drive to eat or simply affect physical self-sufficiency and the ability to procure and prepare food. Patients who are malnourished at hospital discharge have a significantly higher risk of readmission [36,37], and the immediate 30-day post-hospital period is particularly vulnerable as approximately 20 percent of older patients are readmitted to hospital due to reasons other than the initial diagnosis (“post-hospital syndrome”) [38]. Patients at discharge with severe weight loss as well as fatigue are at double-risk of readmission and impaired outcome and are thus in higher need of increased medical attention and multimodal treatment in the ambulatory setting. Our findings contribute to the recognition of fatigue as a frequent condition in the old, which unfortunately is still neglected in the health care system. Additionally, we show a link between fatigue and the nutritional state although more information is needed to elucidate the true association between macro- and micronutrient deficiency and the development of fatigue. Fatigue has a predictive character [10], and it may be a potential self-reported predictor of early frailty and precursor of disability where disability does not exist yet. Therefore, identification of both fatigue and nutritional status including weight loss, as well as fatigue-related impairments, are necessary at admission to the hospital and at hospital discharge to allow for treatment in the hospital but also in the community setting. More research is, however, needed on the assessment of fatigue, its treatment, and the effect of medical or nutritional intervention on fatigue. A multimodal intervention including both exercise and dietary intervention is most likely needed to address the complex and multifactorial syndrome of fatigue. 

In summary, our data imply that fatigue not only results from disease, age-related physiological changes, such as altered neuromuscular function or immobility, cognitive impairment, low-grade inflammation, pain [39], and psychological parameters (e.g., mood disorders such as depression) but is most probably affected by nutritional status as well. 

This study has some limitations that should be noted. Apart from the cross-sectional study design which does not allow conclusions on cause and effect, a healthy age-matched control group in whom fatigue has a different character [40] would have been interesting to further study the relationship between nutritional status and fatigue. Additionally, the use of self-reported weight loss, which although routinely used, may be subject to recall error.

## 5. Conclusions

Our study results show that old patients with self-reported severe weight loss experienced significantly higher fatigue levels. The degree of self-reported weight loss in the old predicted fatigue severity. Given the established high risk of impaired outcome and readmission in patients with weight loss and fatigue, assessing as well as treating both malnutrition and fatigue on admission as well as on discharge are recommended to improve post-hospital outcomes. 

## Figures and Tables

**Table 1:** Demographic, clinical characteristics, nutritional, and functional parameters of all recruited patients at hospital discharge and classified according to self-reported severe weight loss.

Parameter	All Patients *n* = 424	Patients with No Weight Loss or < 5%/3 mo *n* = 284	Patients with Severe Weight Loss *n* = 140	* *p*
**Age (years)**	77.9 ± 6.8	78.7 ± 6.6	76.7 ± 7.0	0.004
**Sex (men/women, %)**	40.1/59.9	36.9/63.1	45.7/54.3	0.084
**BMI (kg/m^2^)**	25.4 ± 5.3	25.8 ± 5.1	24.8 ± 5.7	0.062
**Activities of daily living (score)**	83.1 ± 19.3	84.9 ± 17.6	79.6 ± 22.2	0.01
**Isometric handgrip strength (kg)**	22.9 ± 8.2	23.2 ± 8.1	22.3 ± 8.4	0.285
**Gait speed (cm/s)**	69.9 ± 26.1	71.7 ± 26.9	66.7 ± 24.5	0.103
**Age ≤ 65 y**	*n* = 16	*n* = 10	*n* = 6	
**Type of principal diagnosis (%) †**				
*Orthopedic*	40.0	44.4	33.3	
*Cardiac*	20.0	22.2	16.7	
*Oncologic*	20.0	22.2	16.7	
*Neurologic*	0	0	0	
*Pulmonary*	6.7	0	16.7	
*Gastrointestinal*	13.3	11.1	16.7	
*Renal*	0	0	0	
*Other diseases*	0	0	0	
**Number of comorbidities (*n*)**	7.6 ± 4.0	7.7 ± 4.1	7.5 ± 4.3	
**Number of medications (drugs/day)**	10.2 ± 3.4	10.8 ± 3.5	9.8 ± 3.5	
**Length of hospital stay (d)**	22.2 ± 5.3	21.3 ± 4.3	23.3 ± 7.2	
**Age > 65 y**	*n* = 408	*n* = 274	*n* = 134	
**Type of principal diagnosis (%)**				<0.001
*Orthopedic*	44.2	50.4	32.1	
*Cardiac*	16.5	15.8	17.9	
*Oncologic*	7.4	5.0	11.9	
*Neurologic*	8.6	10.4	5.2	
*Pulmonary*	6.9	6.5	7.5	
*Gastrointestinal*	6.6	5.4	9.0	
*Renal*	3.6	2.7	5.2	
*Other diseases*	6.3	3.8	11.2	
**Number of comorbidities (*n*)**	6.8 ± 3.8	6.7 ± 3.9	7.1 ± 3.5	0.243
**Number of medications (drugs/day)**	9.9 ± 3.8	9.4 ± 3.8	10.9 ± 3.6	<0.001
**Length of hospital stay (d)**	19.3 ± 4.8	18.7 ± 4.4	20.1 ± 5.4	0.006

Values are presented in mean ± SD or categories in relative numbers (percentage). * *p* values were calculated between the patient groups (*p* < 0.05). † Comparisons were not made in the age group < 65 y due to small sample size. Abbreviations: body mass index (BMI).

**Table 2:** Fatigue in all recruited patients and stratified according to self-reported severe weight loss.

BFI Fatigue Items	All Patients *n* = 424	Patients with No Weight Loss or < 5%/3 mo *n* = 284	Patients with Severe Weight Loss *n* = 140	* *p*
**Fatigue right now (score)**	3.9 ± 2.6	3.8 ± 2.5	4.2 ± 2.6	0.15
**Usual fatigue (score)**	4.2 ± 2.5	4.0 ± 2.4	4.5 ± 2.5	0.028
**Worst fatigue (score)**	5.2 ± 2.9	5.0 ± 2.9	5.7 ± 2.8	0.038
**Fatigue-related impairment (score)**				
*Activity*	3.0 ± 3.1	2.8 ± 3.0	3.4 ± 3.2	0.062
*Mood*	2.6 ± 3.0	2.4 ± 2.8	2.9 ± 3.2	0.111
*Walking ability*	3.5 ± 3.2	3.3 ± 3.1	3.9 ± 3.2	0.08
*Work*	3.2 ± 3.3	3.0 ± 3.2	3.6 ± 3.4	0.133
*Relationships with others*	2.0 ± 2.7	1.8 ± 2.5	2.3 ± 3.0	0.063
*Vitality*	2.5 ± 2.9	2.3 ± 2.7	2.9 ± 3.3	0.054

Values are presented in mean ± SD. * *p* values were calculated between the patient groups (*p* < 0.05).

**Table 3:** Risk factors for severity of fatigue identified by multinomial regression analyses in patients with self-reported weight loss (model 1).

Parameter	OR	95 % CI	*p*
**Mild fatigue**			
Weight loss in the last 3 months (%)	1.109	0.975;1.262	0.115
**Moderate fatigue**			
Weight loss in the last 3 months (%)	1.172	1.026;1.338	0.019
**Severe fatigue**			
Number of medications (drugs/day)	1.22	1.023;1.455	0.027
Weight loss in the last 3 months (%)	1.209	1.047;1.395	0.01

*n* = 278. Multinomial regression (model 1) adjusted for age, sex, number of comorbidities, and number of medications as well as BMI. Dependent reference variable: no fatigue. Abbreviation: confidence interval (CI), odds ratio (OR).

**Table 4:** Risk factors for moderate/severe fatigue identified by binary logistic regression analyses in the total number of patients (model 2).

Parameter	OR	95 % CI	*p*
**Age (years)**	1.027	0.994;1.060	0.111
Male sex *	1.190	0.771;1.837	0.433
**Number of comorbidities**	0.978	0.920;1.040	0.480
**Number of medications (drugs/day)**	1.057	0.993;1.125	0.082
**BMI (kg/m^2^)**	1.036	0.995;1.080	0.086
Self-reported severe weight loss **	1.651	1.052;2.590	0.029

*n* = 424. Dependent reference variable: no/mild fatigue. * compared to female sex, ** compared to patients with no weight loss or < 5%/3 mo. Abbreviations: body mass index (BMI), confidence interval (CI), odds ratio (OR).
